# Fluorescent glycan fingerprinting of SARS2 spike proteins

**DOI:** 10.1038/s41598-021-98919-4

**Published:** 2021-10-14

**Authors:** Zhengliang L. Wu, James M. Ertelt

**Affiliations:** Bio-techne, R&D Systems, Inc., 614 McKinley Place N.E., Minneapolis, MN 55413 USA

**Keywords:** Glycoconjugates, Glycobiology

## Abstract

Glycosylation is the most common post-translational modification and has myriad of biological functions. However, glycan analysis has always been a challenge. Here, we would like to present new techniques for glycan fingerprinting based on enzymatic fluorescent labeling and gel electrophoresis. The method is illustrated on SARS2 spike (S) glycoproteins. SARS2, a novel coronavirus and the causative agent of the COVID-19 pandemic, has had significant social and economic impacts since the end of 2019. To obtain the N-glycan fingerprint of an S protein, glycans released from the protein are first labeled through enzymatic incorporation of fluorophore-conjugated sialic acid or fucose, then separated by SDS-PAGE, and finally visualized with a fluorescent imager. To identify the labeled glycans of a fingerprint, glycan standards and glycan ladders are enzymatically generated and run alongside the samples as references. By comparing the mobility of a labeled glycan to that of a glycan standard, the identity of glycans maybe determined. O-glycans can also be fingerprinted. Due to the lack of an enzyme for broad O-glycan release, O-glycans on the S protein can be labeled with fluorescent sialic acid and digested with trypsin to obtain labeled glycan peptides that are then separated by gel electrophoresis. Glycan fingerprinting could serve as a quick method for globally assessing the glycosylation of a specific glycoprotein.

## Introduction

Glycosylation is the most common post-translational modification. Different glycan patterns can increase protein stability, solubility, and affect cell–cell interactions^1,2^. Glycans also play various roles in immunity, such as modulating T cell activation, leukocyte homing, and the immunogenicity of proteins^3,4^. Aberrant glycosylation is believed to be one of the causes for autoimmune diseases^5^. Taking advantage of their biological functions, many viral pathogens hijack the glycosylation machinery of host cells to alter their own physiological properties and evade host immune surveillance^6–8^.

Glycan analysis is critical for researchers to better understand the biological functions of glycoproteins. Current methods for glycan analysis include mass spectrometry, liquid chromatography, and others^9^. However, mass spectrometry is expensive and requires expertise, making it difficult for many laboratories to perform. In addition, the data obtained can show variation among laboratories, even when studying the same glycoprotein, likely due to using different machines and different ways of sample process. For example, the NIST Monoclonal Antibody Reference Material 8671 has been analyzed by 66 labs around the world and the diversity of the results was large, with the number of glycan compositions identified by each laboratory ranging from 4 to 48^10^. Therefore, additional methods for glycan study that can be conveniently performed in common laboratories maybe beneficial.

Since the end of 2019, the severe acute respiratory syndrome coronavirus-2 (SARS-CoV-2) pandemic has become a major threat to human health^11^. The virus utilizes its membrane spike (S) glycoprotein to bind to angiotensin-converting enzyme 2 (ACE-2) to initiate the invasion of host cells^12,13^. The S protein is proteolytically cleaved by furin into two functional subunits, S1 and S2. S1 contains a receptor binding domain (RBD) and is responsible for the initial attachment of the virus to the surface of host cells and S2 is responsible for membrane fusion that triggers entry of the virus into the host cells^14^. The S protein contains 22 N-glycan sequons (N-X-S/T motifs, where X is any amino acid except proline) and it is believed that glycosylation serves to camouflage the immunogenic epitopes of the protein, enhancing the virus’s ability to evade the immune response^15^. In addition, the glycans directly modulate the spike-ACE2 interactions^16^. In turn, the S protein is the principal target for vaccine development and a critical component of serological assays^17–19^. To enhance the success of vaccination and serological testing, understanding protein glycosylation is critical. Previously, mass spectrometry analysis revealed that the spike protein is highly modified with complex, hybrid, oligomannose N-glycans and more complicated N- and O-glycans^15,20–22^; however, the glycan profiles reported varied greatly. For example, while Watanabe et al. reported that only complex types of N-glycans were found on the RBD domain, Shajahan et al. and Zhou et al*.* showed that oligomannose was the major glycan found. In addition, Shajahan et al*.* reported the presence of O-glycans on the RBD domain and Sanda et al. reported the presence of O-glycans near the furin cleavage site. O-glycans were not reported by Watanabe et al. and Zhou et al*.* These discrepancies could be due to the de facto variations on the glycosylations of the various recombinant spike proteins and different mass spectrometry methods employed in those studies, but also highlight the necessity for additional methods of glycan verification.

**Figure 1:**
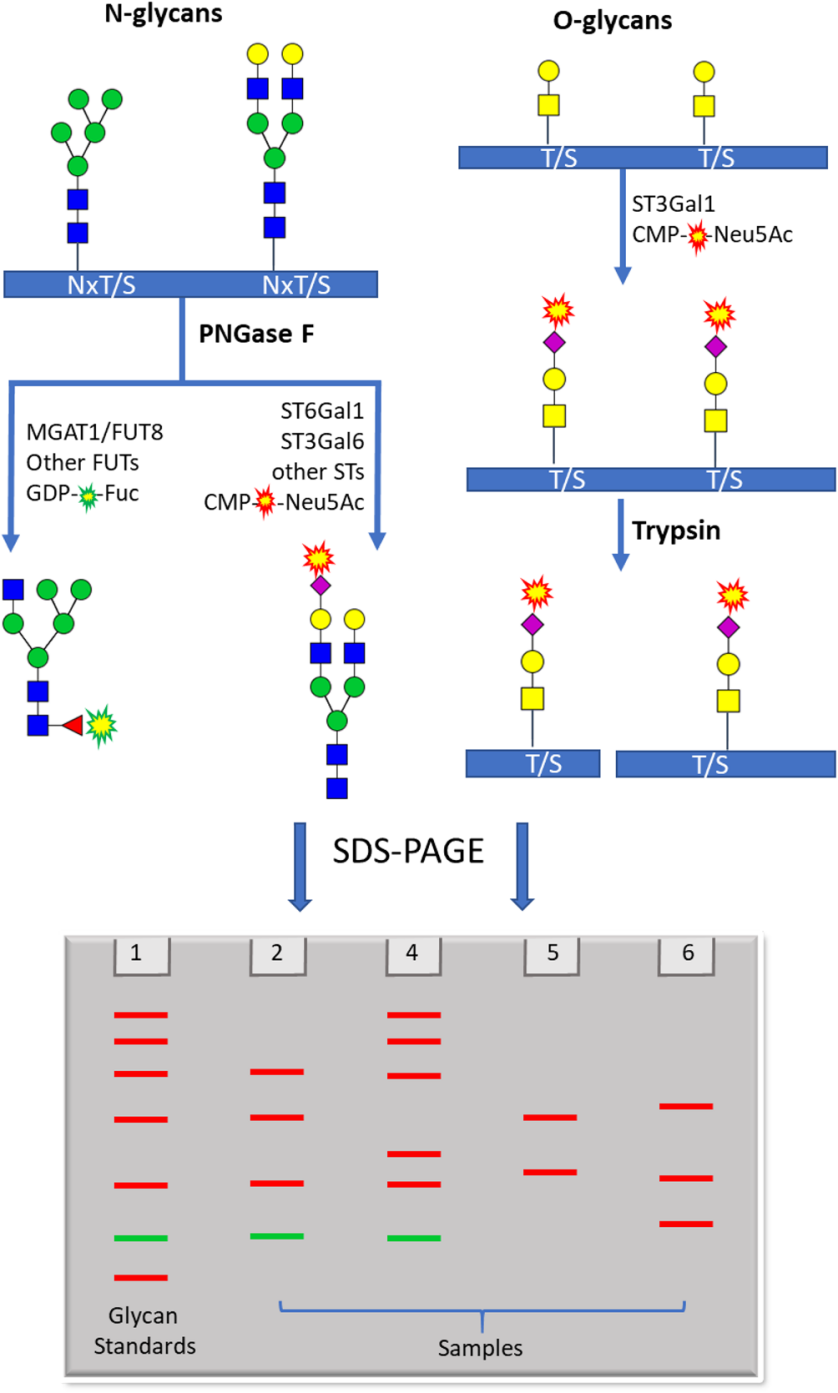
Flow chart for glycan finger-printing. N-glycans are released by PNGase F and then labeled by fluorescent fucose or sialic acid through different glycosyltransferases individually or in combination. O-glycans are labeled with O-glycan specific sialyltransferases and then digested with trypsin to obtain glycopeptides of different sizes. The labeled N-glycans and O-glycan peptides are then separated on SDS-PAGE. The identities of separated N-glycans maybe deduced by comparing with glycan standards running along with.

Previously we reported the separation of fluorophore tagged N-glycans released from fetal bovine fetuin and some pharmaceutical antibodies by sodium dodecyl sulfate polyacrylamide gel electrophoresis (SDS-PAGE)^23^. Further in this direction, we would like to present a novel glycan fingerprinting technique based on non-reducing labeling for studying glycosylation (Fig. 1), which is different from capillary electrophoresis of fluorescent glycans labeled at the reducing ends^24,25^. In our method, N-glycans are first released by peptide N-glycosidase F (PNGase F) treatment, with or without additional sialidase and fucosidase. This is followed by labeling with fluorophore-conjugated sialic acid or fucose using a sialyltransferase or a fucosyltransferase, respectively^23,26^. O-glycans are labeled first, then the glycoprotein is digested by trypsin, and finally the glycopeptides are separated by SDS-PAGE. These methods allow for quick assessment of the glycosylation pattern of a glycoprotein. As an example, we applied these techniques to study the glycosylation of several SARS2 S protein constructs expressed from different host cells.

## Results

### Electrophoretic mobility of Cy5-labeled glycans on SDS PAGE

**Figure 2:**
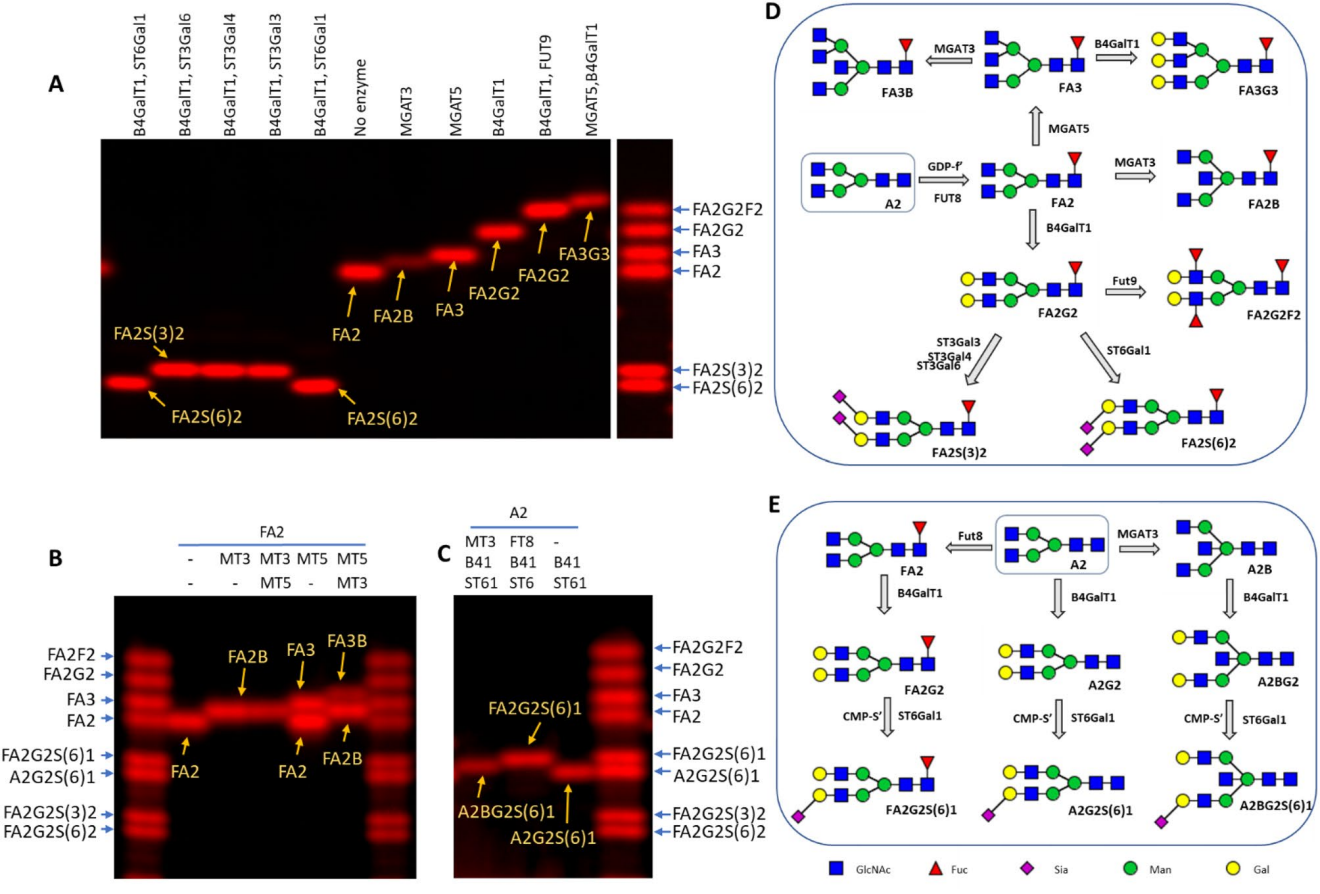
Relative mobilities of various Cy5-labeled glycans and their enzymatic synthesis. All glycans were enzymatically synthesized starting from the glycan A2 (known as G0 in IgG glycan notation). The Glycan ladders were composed by combining some of the labeled glycans. With the exception of the enzymatic reactions of FUT8, MGAT3 and MGAT5, all other enzymatic reactions resulted in one or two intermediates as there are two or more branches in each glycan that allow enzymatic modification (see Supplemental Fig. 1 for intermediates). The labeled glycans were separated on a 17% gel and visualized with a fluorescent imager. (**A**) Relative mobilities of glycans that were labeled at the core-fucose. The enzymes used for generating these glycans are listed on the top of the image. (**B**) Relative mobility change on FA2 by addition of a bisecting GlcNAc introduced by MGAT3 (MT3) versus a β1-6GlcNAc introduced by MGAT5 (MT5). MGAT3 and MGAT5 were introduced in different orders. The 2nd enzyme was introduced 30 min after the 1st enzyme. FA3B containing both a bisecting and a β1-6 GlcNAc can only be observed when MGAT5 was introduced first. (**C**) Relative mobility change on A2G2S(6)1 by addition of a bisecting GlcNAc versus a core-fucose. The glycans were generated and labeled starting from A2 with the enzymes indicated at the top of the gel in the specified order. MT3, MGAT3; FT8, FUT8; B41, B4GalT1; ST61, ST6Gal1. (**D**) Schemes for enzymatic generation of the labeled glycans in (**A**) and (**B**). Labeling was through FUT8 and GDP-Cy5-Fuc (GDP-f’). (**E**) Schemes for enzymatic generation of the labeled glycans in (**C**). Labeling was through ST6Gal1 and CMP-Cy5-Neu5Ac (CMP-S’). Two Cy5-Neu5Ac residues can be introduced to each of the glycans, but only glycans with one Cy5-Neu5Ac were displayed in (**B**), (**C**) and (**E**).

To correlate the glycan structures to their mobility, we established a series of labeled glycans based on a biantennary antibody glycan A2 (Fig. 2). The short names for glycans in this study are based on Oxford Glycan Notation (Table 1). A2 was first labeled by FUT8 with Cy5-conjugated fucose to become FA2. A series of glycans were then generated enzymatically based on FA2 (Fig. 2A,B). A2 was also extended by B4GalT1, with or without prior modification by FUT8 and MGAT3, and finally labeled by ST6Gal1 with Cy5-Neu5Ac to generate FA2G2S(6)1, A2BG2S(6)1 and A2G2S(6)1 (Fig. 2C). The following observations were made regarding the mobility change caused by the addition of different monosaccharides. First, addition of a neutral monosaccharide such as a Gal, GlcNAc, and Fuc to a glycan slows down the mobility of the glycan at a noticeable rate. Second, addition of a bisecting GlcNAc slows down the mobility of a glycan at roughly half the rate of that of a β,6-linked GlcNAc. Third, addition of a sialic acid residue significantly increases the mobility of a glycan, with α,6-linked sialic acid exhibiting greater mobility than α,3-linked sialic acid. Likewise, when a monosaccharide is added at multiple positions on a glycan, intermediate glycosylation products exhibit intermediate mobilities. For example, FA2G1 moves faster than FA2G2, and FA2G2S1 moves slower than FA2G2S2 (Supplemental Fig. 1). Intermediate products were only observed within a short time window and were converted to final products after prolonged incubation. The contributions of various monosaccharides to gel mobility are summarized in Table 2.

**Table 1:**
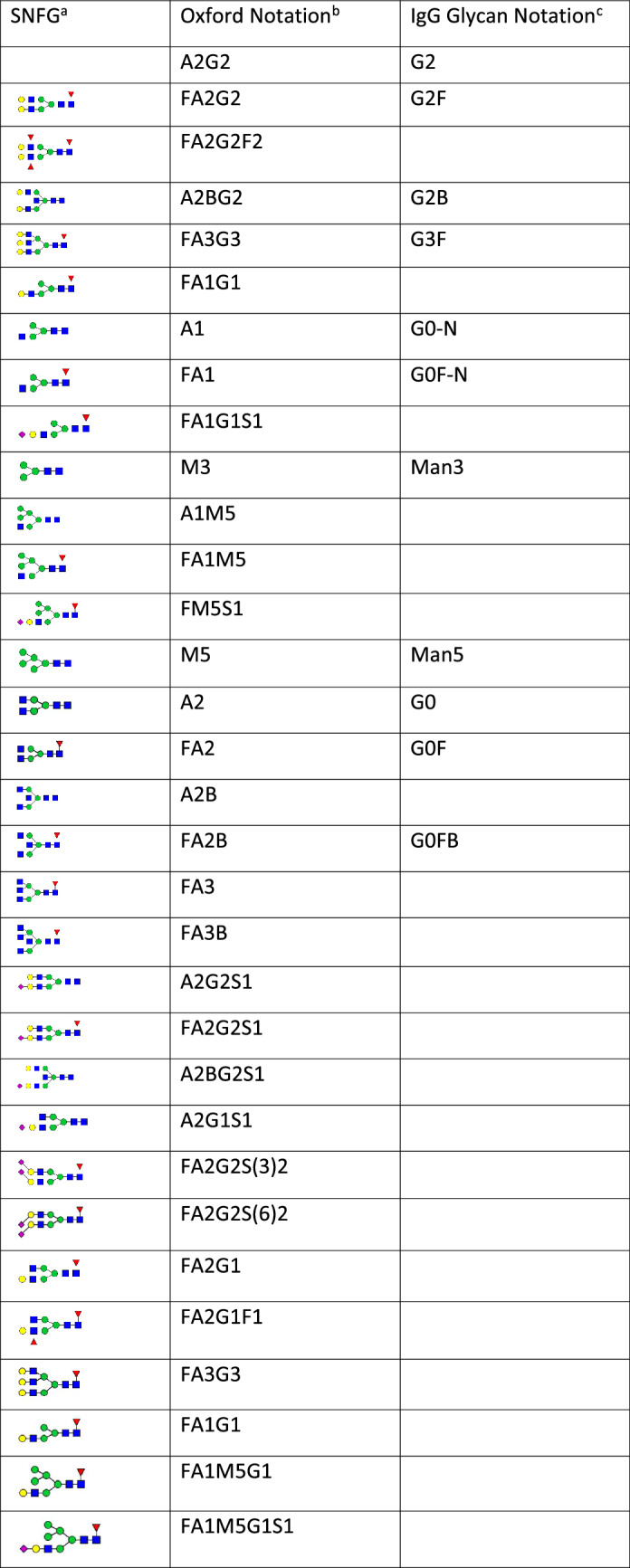
Symbol and short names of the glycans mentioned in this report.

^a^SNFG stands for Symbol Nomenclature for Glycan.

^b^Rules of abbreviation: front F, core fucose; end F, fucose on antenna; M, mannose; A, antenna; G, galactose; S, sialic acid; abbreviations are followed by numbers of sugar residue or antennae; a number in a bracket immediately after an abbreviation indicates the linkage specificity of the sugar residue. If not specified, G refers to G(4), S refers to S(6). M3 and G2 are common to many complex glycans and maybe omitted from the short names. For examples; A1M3 is shorted as A1, and FA2G2S(3)2 maybe shorted as FA2S(3)2.

^c^This is primarily for indicating the presence of core fucose, and the number of galactoses that are present on biantennary Ig G glycans.

**Table 2:** Contributions of various monosaccharides to relative mobility (rm).

Glycans and their rm		Contributions of individual monosaccharides to rm			
Glycans	rm	Pairs of glycans	difference by monosaccharides	Δrm	Δrm/monosaccharide
FA2G2F2	− 0.521	FA2G2F2, FA2G2	2 × Fuc	− 0.185	− 0.092/Fuc
FA2G2	− 0.336	FA2G2, FA2	2 × Gal	− 0.336	− 0.168/Gal
FA3B	− 0.240	FA3B, FA3	1 × b-GlcNAc	− 0.087	− 0.087/b-GlcNAc
FA3	− 0.154	FA3, FA2	1 × GlcNAc	− 0.154	− 0.154/GlcNAc
FA2B	− 0.092	FA2B, FA2	1 × b-GlcNAc	− 0.092	− 0.092/b-GlcNAc
FA2	0.000				
FA2G2S(6)1	0.381	FA2G2S(6)1, A2G2S(6)1	1 × core-Fuc	− 0.119	− 0.119/core-Fuc
A2G2S(6)1	0.501				
FA2G2S(3)2	0.894	FA2G2S(3)2, FA2G2	2 × α3-Sia	1.231	0.615/α3-Sia
FA2G2S(6)2	1.000	FA2G2S(6)2, FA2G2	2 × α6-Sia	1.336	0.676/α6-Sia

The rm of each individual glycan was determined based on the gel images in Fig. 2, where the relative mobility of FA2 was arbitrarily set to 0 and that of FA2G2S(6)2 was set to 1. The contribution of a monosaccharide to the mobility was calculated based on the difference in the mobility of a pair of glycans that differed only by the monosaccharide. A minus number indicates that the monosaccharide slows down the mobility and a positive number indicates that the monosaccharide makes the mobility faster*. *

*b-GlcNAc* bisecting-GlcNAc.

### Selection of labeling enzyme and optimization of substrate concentrations

Before fingerprinting glycans released from various SARS2 spike proteins, we screened the labeling enzymes and optimized the substrate concentration for the labeling reaction using glycans released from the RBD protein expressed in CHO cells. The glycans were first probed by various sialyltransferases, including ST6Gal1, which generates α2,6-sialylated N-glycans^27^, and, ST3Gal3, ST3Gal4 and ST3Gal6, which generate α2,3-sialylated N-glycans^28,29^. Among these enzymes, ST6Gal1 and ST3Gal6 gave stronger signals (Supplemental Fig. 2A) and were chosen for the following glycan fingerprinting study. Stronger signal intensities were also observed when the substrate input was around 2 µg (Supplemental Fig. 2B) and the donor CMP-Cy5-Neu5Ac input was around 0.4 nmol (Supplemental Fig. 2C), therefore, these conditions were chosen for the following fingerprinting study.

### N-glycan fingerprinting study of SARS2 spike proteins with ST6Gal1

**Figure 3:**
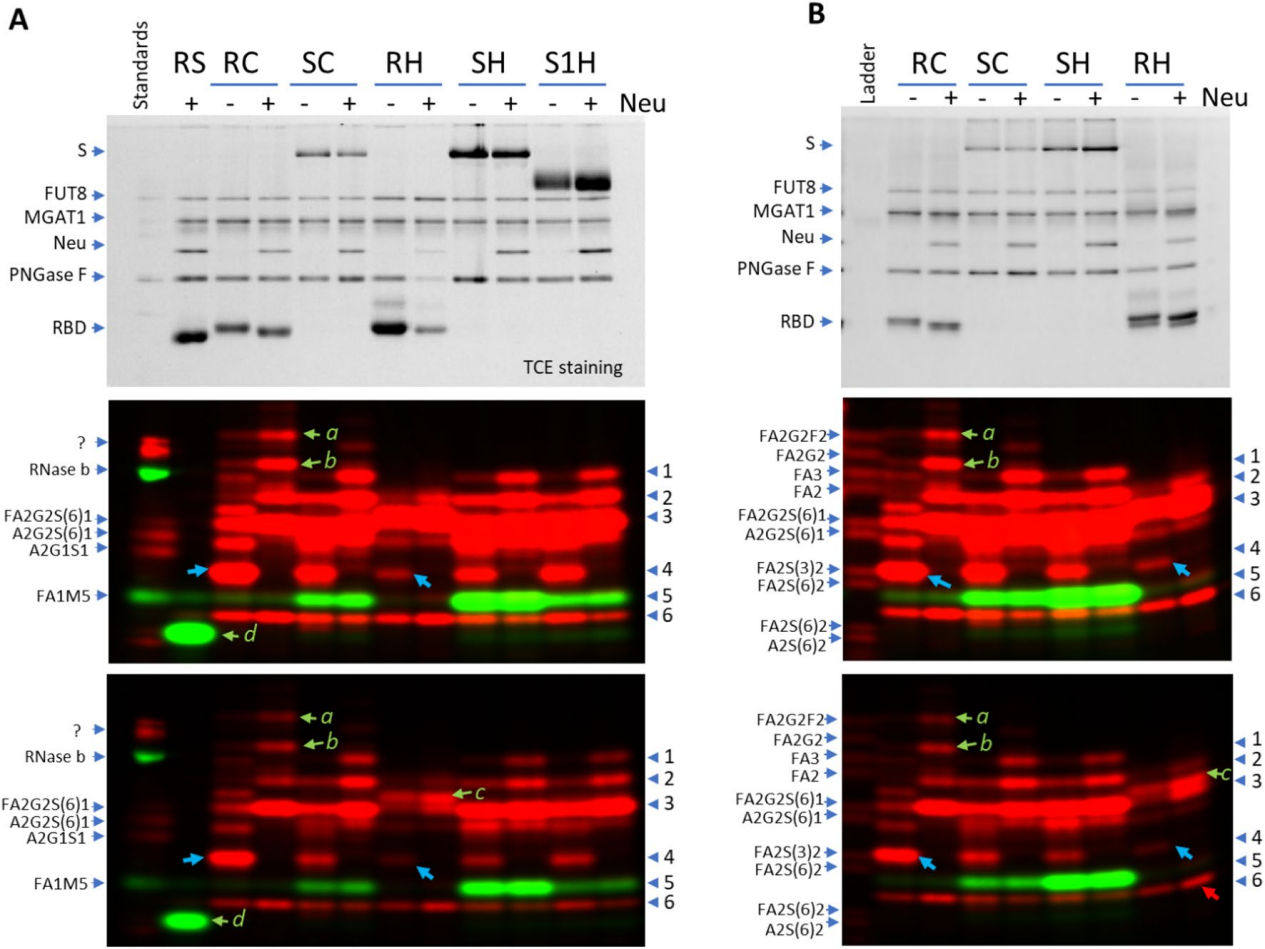
Fingerprinting N-glycans released from various SARS2 Spike proteins with ST6Gal1 (red) and FUT8 (green). N-glycans of various recombinant spike proteins released by PNGase F were labeled by ST6Gal1 and FUT8 with (+) or without (−) pretreatment of a neuraminidase (Neu). Labeled samples were separated on a 17% SDS-PAGE gel and imaged with traditional protein imaging (upper panels) or fluorescent imaging (middle and lower panel in different contrasts). Bands in red are complex or hybrid glycans labeled by ST6Gal1 (via the incorporation of Cy5-Neu5Ac). Bands in green are oligo-mannose glycans labeled by FUT8 (via the incorporation of Alexa Fluor 555-conjugated fucose). For FUT8 labeling, MGAT1 and UDP-GlcNAc were also included in the labeling mix to convert the oligo-mannose glycans to the substrates for FUT8. Labeled glycans from ribonuclease B, A2G1S1, A2G2S(6)1 and FA2G2S(6)1 were run as references in (**A**) and a glycan ladder with 10 labeled standard glycans was run in (**B**). RS, RBD domain of SARS2 Spike protein expressed in *Sf*21 cells; RC, RBD domain of SARS2 Spike protein expressed in CHO cells; RH, RBD domain of SARS2 Spike protein expressed in HEK293 cells; SC, whole SARS2 Spike protein expressed in CHO cells; S1H, SARS2 S1 protein expressed in HEK293 cells; SH, SARS2 Spike protein expressed in HEK293 cells.

N-glycans released from the following SARS2 spike protein constructs, with or without prior desialylation, were labeled with ST6Gal1/CMP-Cy5-Neu5Ac: RBD domain expressed in *Sf*21 cells (RS), RBD domain expressed in CHO cells (RC), RBD domain expressed in HEK293 cells (RH), full length spike protein expressed in CHO cells (SC), full length spike protein expressed in HEK293 cells (SH), and S1 protein expressed in HEK293 cells (S1H). As the presence of oligomannose glycans on S proteins were reported previously^15,21^, FUT8/GDP-Alexa Fluor 555-Fuc together with MGAT1/UDP-GlcNAc, that allows the labeling of M3 and M5 (commonly known as Man3 and Man5)^23^ were also added to the final labeling reactions to reveal these glycans. ST6Gal1 labeling revealed a series of bands with large variations from all constructs except RS (Fig. 3A). In general, desialylation resulted in elimination of some fast-moving bands and increased labeling on some slow-moving bands, suggesting the existence of both sialylated and asialylated glycans on these proteins.

Several common bands were observed (band 1–6) in Fig. 3. Bands 1 and 2 were mainly found in neuraminidase treated SC, SH and S1H at the position around FA3 and FA2 (Fig. 3B). Since labeling through ST6Gal1 also contributes a sialic acid and therefore makes a labeled glycan move much faster, band 1 and band 2 could be due to labeling on highly branched complexed glycans, such as tetra- and tri-antennary complex glycans. Band 1 was mainly observed in desialylated SC, SH and S1H, suggesting that the glycan was initially sialylated. Band 2 was observed in SC, SH and S1H samples before and after desialylation, but displayed a significant signal increase upon desialylation, suggesting that the glycan was initially largely sialylated. Band 3 was prominent in all SH and S1H samples and in desialylated samples of RC and SC and had the same mobility of FA2G2S(6)1 (Fig. 3B). Since band 3 and the reference glycan FA2G2S(6)1 had the same labeling (Cy5-Neu5Ac) and same mobility, band 3 is likely to be FA2G2S(6)1 and was the labeling product of FA2G2 (Fig. 2E). The fact that band 3 was much weaker in RC and SC than in desialylated RC and SC samples suggests that the glycan was initially sialylated in these samples. Opposite to band 3, band 4 around the position of FA2G2S(3)2 had a strong presence in RC and SC but not in the desialylated RC and SC samples, suggesting that band 4 was due to the labeling of a partially sialylated glycan that was converted to band 3 when desialyation occurred before labeling. Band 5 was likely due to the labeling of oligomannose M5 as the band had the same mobility as the reference glycan, FA1M5 (Fig. 3A). Band 6 had almost equal intensity in all samples and did not respond to *C.perfringens* Neuraminidase treatment. The fast mobility of band 6 suggests that it is highly sialylated, but the fact that it was unresponsive to neuraminidase treatment suggests the opposite. The nature of band 6 remains to be investigated.

Most of the common bands displayed great variation among the samples. For example, band 4 was the most abundant in RC, but almost at negligible level in RH (blue arrows in Fig. 3); band 5 was the most abundant in SH, but almost completely lacking in RH. Surprisingly, some bands were only found in one sample but not the others, such as bands *a*, *b*, *c* and *d* (Fig. 3A). Band *a* and *b* in RC had slow mobility and responded to neuraminidase treatment, suggesting that they had highly complexed structures and were initially sialylated. Band *c* in RH was just above the position of FA2G2S(6)1, suggesting that it might be FA2BG2S(6)1 that contains a bisecting GlcNAc (Fig. 2B,C). Band *d* labeled by FUT8 was found only in RS and had faster mobility than FA1M5, suggesting that it might be FA1 (labeled product of M3), consistent with the notion that M3 is a main glycan expressed in insect cells^30^. Additional enzymatic conversion of band *d* with B4GalT1 and ST6Gal1 further confirmed the identity of band *d* (Supplemental Fig. 4).

### N-glycan fingerprinting study of SARS2 spike proteins with ST3Gal6

**Figure 4:**
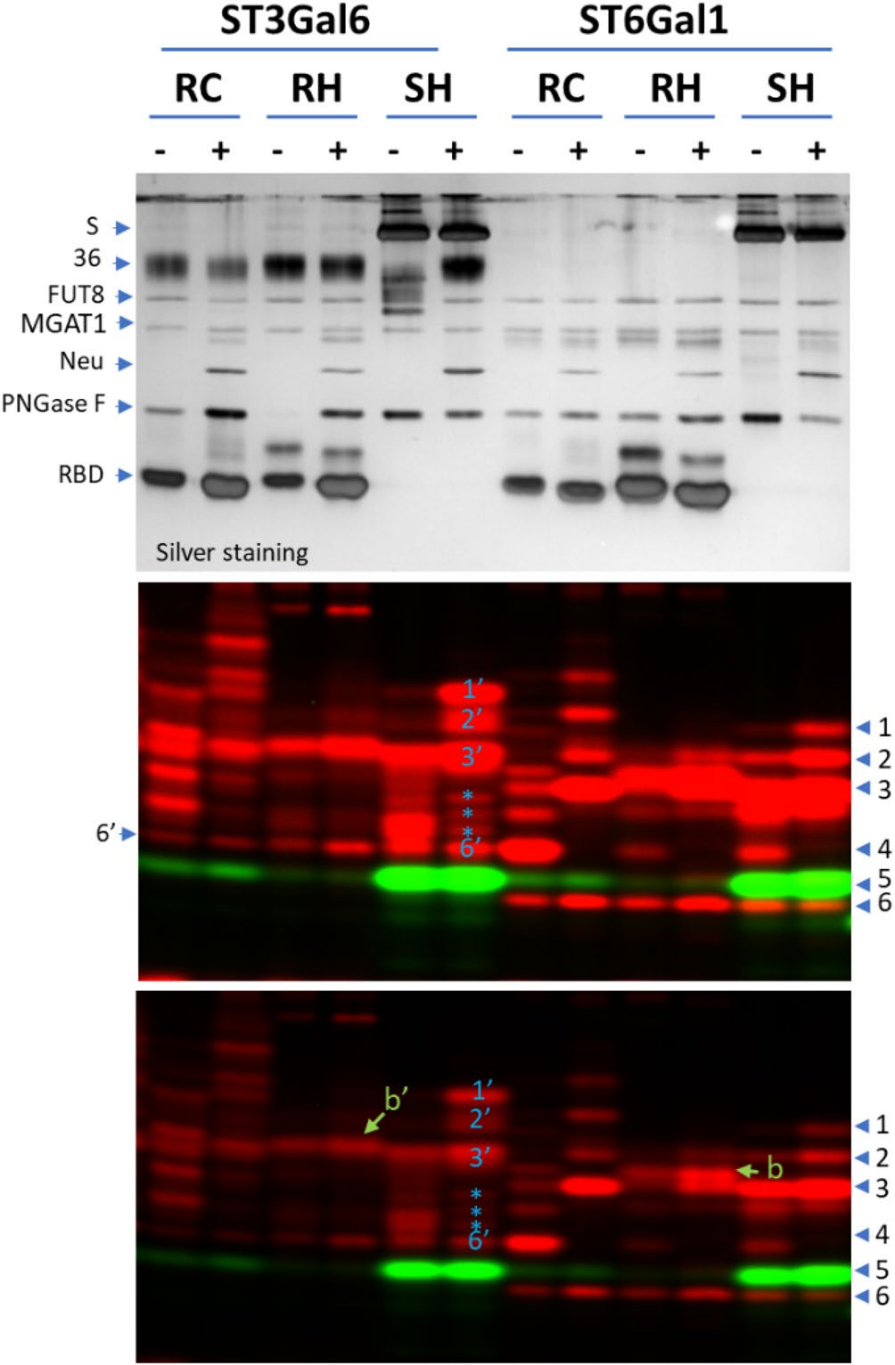
Fingerprinting by ST3Gal6 and ST6Gal1 on various SARS2 Spike proteins. Glycans of various recombinant SARS2 spike proteins released by PNGase F with (+) or without (−) neuraminidase pretreatment were labeled by ST3Gal6 or ST6Gal1 together with FUT8 and separated on a 17% SDS gel. The gel was then imaged with silver staining (upper panel) or fluorescent imaging (middle and lower panel in different contrasts). Bands in red are complex or hybrid glycans labeled by ST6Gal1 or ST3Gal6 (via the incorporation of Cy5-Neu5Ac). Bands in green are oligo-mannose glycans labeled by FUT8 (via the incorporation of Alexa Fluor 555-conjugated Fucose). For oligo-mannose labeling, MGAT1 and UDP-GlcNAc were also included in the labeling mix to convert the glycans to the substrates for FUT8. RC, RBD domain of the SARS2 Spike protein expressed in CHO cells; RH, RBD domain of the SARS2 Spike protein expressed in HEK293 cells; SH, whole SARS2 Spike protein expressed in HEK293 cells.

Both ST6Gal1 and ST3Gal6 are known to sialylate the Galβ1,4GlcNAc structure on glycoproteins^29^. When the same set of SARS2 spike protein samples were probed with ST3Gal6, similar but distinctive glycan fingerprints were observed (Fig. 4). It seems that the entire banding pattern revealed by ST3Gal6 was shifted up from that of ST6Gal1. For example, bands 1′, 2′, 3′, and 6′ in the ST3Gal6 labeled SH sample corresponded well with bands 1, 2, 3, and 6 in the ST6Gal1 labeled SH sample; similar to band 6, band 6′ was found across all lanes; similar to the relative positioning of band *b* and band 3 revealed by ST6Gal1, band *b*′ labeled by ST3Gal6 was shifted slightly up from band 3′. The shift observed in the bands revealed by ST3Gal6 compared to those in the ST6Gal1 labeled sample is likely because glycans with an α,3-linked sialic acid had slower mobility than corresponding glycans with α,6-linked sialic acid (Fig. 2A, Table 2).

While there was similarity between the fingerprints revealed by the two enzymes, ST3Gal6 labeling also revealed some unique bands. For example, bands marked with asterisks in SH revealed by ST3Gal6 had no corresponding bands in SH revealed by ST6Gal1. This difference in the banding pattern suggests that ST3Gal6 and ST6Gal1 have overlapping but distinct substrate preferences.

### Fingerprinting O-glycans of SARS2 spike proteins with ST3Gal1

O-glycosylation of the RBD domain of the SARS-Cov-2 spike protein has been reported previously^21,31^. Here, we investigated whether the fingerprinting technique could be applied to study O-glycans as well. One challenge for fingerprinting O-glycans is that there is no enzyme (corresponding to PNGase F for the removal of N-glycans) that allows for the wide removal of O-glycans. Currently, *E. faecalis* O-glycosidase (Endo EF) is used to remove Core-1, Core-2 and Core-3 type O-glycans^32^. Another challenge is that O-glycans are typically smaller than N-glycans and rather difficult to separate from the donor substrates of various glycosyltransferases by SDS-PAGE.

**Figure 5:**
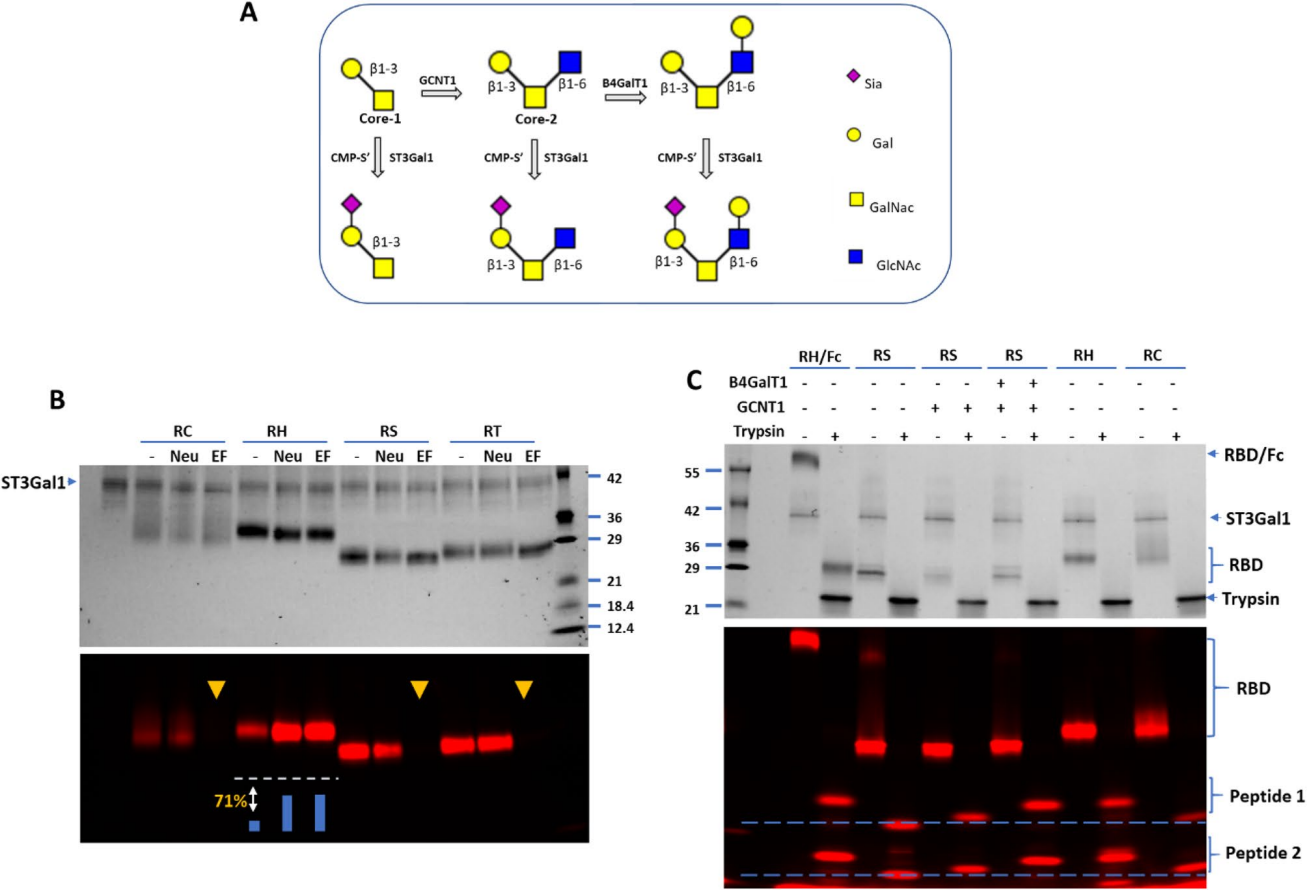
O-Glycan of the RBD domain of SARS-CoV-2 spike protein varies depending on the host cells. (**A**) Strategy for O-glycan conversion and labeling. (**B)** RBD expressed in CHO (RC), HEK293 (RH), *Sf*21(RS) and Tn (RT) cells were probed for O-glycans with CMP-Cy5-Neu5Ac and ST3Gal1. The proteins were directly labeled or labeled after pretreatment with *C. perfringens* neuraminidase alone (Neu) or in combination with Endo EF (EF). Densitometry analysis indicated that neuraminidase treatment resulted in a 71% increase in labeling of the RBD protein expressed in HEK293 cells. (**C**) RBD protein expressed in *Sf*21 cells (RS) pretreated with GCNT1 alone or together with B4GalT1 was labeled with ST3Gal1 and then digested with trypsin. RBD protein fused to the IgG Fc domain expressed in HEK293 cells (RH/Fc), RH and RC samples were also labeled and digested by trypsin and run alongside the RS samples.

To overcome the above challenges, we first directly probed O-glycans on the RBD proteins using O-glycan specific ST3Gal1 as previously described^26^ (Fig. 5A). Indeed, O-glycans were detected on all RBD proteins investigated, but with different levels of sialylation and sensitivity to Endo EF treatment (Fig. 5B). The labeling on the RBD proteins expressed in CHO cells, *Sf*21 cells and Tn cells were removed by Endo EF treatment, further suggesting that the labeled O-glycans were Core-1, Core-2, or Core-3 types. Since Core-3 O-glycan lacks a terminal Gal residue for ST3Gal1 labeling, it was excluded from the possibility. To further identify the O-glycans on the RBD proteins, the RBD protein expressed in *Sf*21 cells was first pretreated with GCNT1^33^, a GlcNAc transferase that converts a Core-1 O-glycan to a Core-2 O-glycan, or together with B4GalT1^34^, which can extend the GlcNAc residue on the Core-2 O-glycan, and then labeled with Cy5-Sialic acid by ST3Gal1, digested with trypsin, and separated in SDS-PAGE (Fig. 5C). Trypsin digestion resulted in two labeled glycopeptides in all samples, but with different mobility. The mobility of the two peptides were shifted by GCNT1 and B4GalT1 sequentially and finally matched to the mobility of the peptides from the RBD protein expressed in HEK293 cells. These results suggest that O-glycans on the RBD protein expressed in HEK293 are extended Core-2 type O-glycans, whereas the O-glycan on the RBD protein expressed in *Sf*21 cells is a Core-1 type.

## Discussion

In this report, we have established a novel method for N-glycan fingerprinting based on enzymatic fluorescent glycan labeling and electrophoresis. As a method of fingerprinting, the overall glycan patterning rather than individual glycan species including their structures is the focus. This method can serve as a quick and inexpensive way to determine if different batches of glycoproteins are consistently glycosylated and identify samples with abnormal glycosylation. Herein, this strategy is demonstrated on several SARS2 spike proteins, in which N-glycans are released and enzymatically labeled with fluorophore-conjugated sialic acid and fucose and separated on SDS-PAGE. Although the strategy does not allow site specific and detailed structural glycan analysis, it does offer some major advantages over traditional similar methods of glycan analysis such as fluorophore-assisted carbohydrate electrophoresis (FACE)^35^. First, it is simpler and more convenient via non-reducing end direct enzymatic labeling. Second, the data acquired can be more informative by simultaneous labeling on different glycans with different fluorophores. Third, multiple samples can be processed simultaneously, therefore it is highly efficient. Fourth, the signal intensity is directly related to the abundance of a glycan species and therefore is more quantitative^23^. Fifth, released glycans and the deglycosylated protein can be viewed in a same gel, allowing strict correlation of a protein and its glycans. While this method only reveals the substrate glycans of the labeling enzyme, and glycans that are not recognized by the labeling enzyme remain undetected, this could be advantageous as well when only specific glycans are being examined.

Recognizing that fingerprinting is focused on general banding patterns, it is always beneficial if the identities of individual bands on a gel can be determined. To this purpose, glycan standards and glycan ladders can be run along with samples to serve as references. By comparing the mobility of labeled bands to the mobility of the reference glycans, the identity of a labeled band maybe inferred. It is also found that the addition of a linkage specific monosaccharide changes the mobility of a glycan at relatively constant rate (Fig. 2; Table 2). The knowledge of mobility shift caused by the addition of certain monosaccharides also allows us to deduce the identities of labeled bands. More specifically, the addition of a neutral monosaccharide slows down a glycan and the addition of a negatively charged sialic acid increases the mobility of a glycan. Particularly, a bisecting GlcNAc causes about half of the mobility shift generated by a β,6-linked GlcNAc and an α,6-linked sialic acid causes slightly more mobility shift than an α,3-linked sialic acid.

Our data suggest that the RBD of the SARS2 spike protein expressed in HEK293 cells mainly contains complex glycans, which is consistent with the reports of Watanabe et al.^6^. Our data also suggests that bisecting GlcNAc may exist on the RBD portion, and oligomannose glycans may mainly exist on the S protein, but not the RBD portion when expressed in HEK293 cells. Our data also indicate that the glycans of the S proteins expressed in insect cells and HEK293 cells are completely different. Using a similar technique, we found that the RBD protein expressed in insect cells contains Core-1 O-glycan, while the RBD protein expressed in HEK293 cells may contain extended Core-2 O-Glycan. Altogether, our study provides evidence to support the claim that host cell determines glycosylation, implying that the glycosylation of SARS2 from COVID-19 patients could be different from those expressed by other hosts, and further suggesting that mRNA vaccines^36^ that involve S antigens produced by recipients themselves but not by other hosts could be more effective on combating COVID-19.

## Material and methods

Different recombinant SARS-CoV-2 Spike RBD proteins expressed in HEK293 cells, Tn5 insect cells, CHO cells, full length recombinant SARS-CoV-2 Spike proteins expressed in HEK293 cells and CHO cells, and recombinant SARS-CoV-2 Spike S1 subunit protein expressed in HEK293 cells were from Bio-Techne. Recombinant human ST6Gal1, FUT8, B4GalT1, MGAT1, ST3Gal6, FUT9, GCNT1, ST3Gal1, ST3Gal4 and ST3Gal3, *C. perfringens* neuraminidase, *F. meningosepticum* PNGase (PNGase F), *E. faecalis* O-Glycosidase (Endo EF), CMP-Cy5-Neu5Ac, GDP-AlexaFluor555-Fuc were from Bio-Techne. IgG glycan G0, G1F and G0F were from Dextra Labs. Trypsin was from Sigma Aldrich.

### Releasing and labeling N-glycans of the spike proteins

To release N-glycans, 5 μg of a spike protein was mixed with 0.2 μg PNGase F and supplemented with labeling buffer (25 mM Tris pH 7.5, 10 mM MnCl_2_) to 20 μL and then incubated at 37 °C for 30 min. For desialylation, an additional 0.2 μg *C. perfringens* neuraminidase was added into the reaction mixture together with PNGase F. The above mixture was then heated at 95 °C for two minutes to inactivate the enzymes. Labeling mixture contained 0.5 μg of a sialyltransferase together with 0.4 nmol of CMP-Cy5-Neu5Ac supplemented with labeling buffer to 10 μL. For labeling oligomannose, an additional 0.5 μg of FUT8 together with 0.4 nmol of GDP-Alexa Fluor 555-conjugated Fuc and 0.5 μg of MGAT1 together with 10 nmol of UDP-GlcNAc were also added into the labeling mixture. The labeling mixture was then added into the reaction mixture and incubated at 37 °C for 2 h or overnight at room temperature.

### Labeling O-glycans of the spike proteins and trypsin digestion

The RBD proteins were labeled with or without pretreatment with *C. perfringens* neuraminidase, Endo EF, GCNT1 and B4GalT1. For pretreatment, 5 μg of a spike protein was mixed with 0.5 μg of the above enzymes individually or in combination (together with 1 mM of UDP-GlcNAc and UDP-Gal in the cases of GCNT1 and B4GalT1, respectively) in 20 μL labeling buffer, and then incubated for 20 min at 37 °C. The samples were then heated at 95 °C for 2 min to inactivate the enzymes. The samples were then labeled by the addition of 0.5 μg ST3Gal1 together with 0.4 nmol of CMP-Cy5-Neu5Ac and incubated at 37 °C for 30 min. The samples were mixed with 2 μg trypsin and incubated at 37 °C for 10 min for trypsin digestion.

### Labeling glycan standards and building glycan ladder

For labeling a glycan with Cy5-Neu5Ac, 1 μg of the standard was mixed with 1 μg of ST6Gal1 and 1 nmol of CMP-Cy5-Neu5Ac together with 0.5 μg B4GalT1 and 10 nmol of UDP-Gal supplemented with labeling buffer to 20 μL and the mixture was then incubated at 37 °C for 2 h or at room temperature for overnight. For labeling a glycan standard with Cy5-Fucose, 2 μg of the standard was mixed with 1 μg of FUT8 and 2 nmol of GDP-Cy5-Fuc supplemented with labeling buffer to 20 μL and the mixture was then incubated at 37 °C for 2 h or at room temperature for overnight. For building a glycan ladder based on Cy5-Fucose labeled glycan standard, 200 ng of the above labeled glycan was extended with one or more of 0.5 μg each of the glycosyltransfeases, including MGAT3, MGAT5, B4GalT1, FUT9, ST3Gal6 and ST6Gal1 together with their donor substrates at 37 °C for 2 h or overnight at room temperature or whenever the reactions were completed. The reactions were then stopped by heating at 95 °C for 2 min. The glycan ladder was built by mixing equal amounts of the above extended labeled glycans.

### Glycan electrophoresis and imaging

All labeled samples including glycan standards were separated on 15% or 17% sodium dodecyl sulfate–polyacrylamide gels at 20 V/cm. After separation, all gels were imaged using a FluorChem M imager (ProteinSimple, Bio-techne). For imaging protein contents, the gel was also imaged with traditional methods such as silver staining or trichloroethanol (TCE) staining.

## Supplementary Information

Supplementary Information.

## Data Availability

All data generated or analyzed during this study are included in this published article (and its Supplementary Information files).

## References

[1] Varki A (2017). Biological roles of glycans. Glycobiology.

[2] Reily C, Stewart TJ, Renfrow MB, Novak J (2019). Glycosylation in health and disease. Nat. Rev. Nephrol..

[3] Baum LG, Cobb BA (2017). The direct and indirect effects of glycans on immune function. Glycobiology.

[4] Marth JD, Grewal PK (2008). Mammalian glycosylation in immunity. Nat. Rev. Immunol..

[5] Maverakis E (2015). Glycans in the immune system and The Altered Glycan Theory of Autoimmunity: A critical review. J. Autoimmun..

[6] Watanabe Y, Bowden TA, Wilson IA, Crispin M (2019). Exploitation of glycosylation in enveloped virus pathobiology. Biochim. Biophys. Acta Gen. Subj..

[7] Vigerust DJ, Shepherd VL (2007). Virus glycosylation: Role in virulence and immune interactions. Trends Microbiol..

[8] Bagdonaite I, Wandall HH (2018). Global aspects of viral glycosylation. Glycobiology.

[9] Marino K, Bones J, Kattla JJ, Rudd PM (2010). A systematic approach to protein glycosylation analysis: A path through the maze. Nat. Chem. Biol..

[10] De Leoz MLA (2020). NIST interlaboratory study on glycosylation analysis of monoclonal antibodies: Comparison of results from diverse analytical methods. Mol. Cell Proteom..

[11] Zhou P (2020). A pneumonia outbreak associated with a new coronavirus of probable bat origin. Nature.

[12] Shang J (2020). Structural basis of receptor recognition by SARS-CoV-2. Nature.

[13] Wang Q (2020). Structural and functional basis of SARS-CoV-2 entry by using human ACE2. Cell.

[14] Walls AC (2020). Structure, function, and antigenicity of the SARS-CoV-2 spike glycoprotein. Cell.

[15] Watanabe Y, Allen JD, Wrapp D, McLellan JS, Crispin M (2020). Site-specific glycan analysis of the SARS-CoV-2 spike. Science.

[16] Zhao P (2020). Virus-receptor interactions of glycosylated SARS-CoV-2 Spike and human ACE2 receptor. Cell Host Microbe.

[17] Pinto D (2020). Cross-neutralization of SARS-CoV-2 by a human monoclonal SARS-CoV antibody. Nature.

[18] van Doremalen N (2020). ChAdOx1 nCoV-19 vaccine prevents SARS-CoV-2 pneumonia in rhesus macaques. Nature.

[19] Gao Q (2020). Development of an inactivated vaccine candidate for SARS-CoV-2. Science.

[20] Sanda M, Morrison L, Goldman R (2021). N- and O-glycosylation of the SARS-CoV-2 spike protein. Anal. Chem..

[21] Shajahan A, Supekar NT, Gleinich AS, Azadi P (2020). Deducing the N- and O-glycosylation profile of the spike protein of novel coronavirus SARS-CoV-2. Glycobiology.

[22] Zhou D, Tian X, Qi R, Peng C, Zhang W (2020). Identification of 22 N-glycosites on spike glycoprotein of SARS-CoV-2 and accessible surface glycopeptide motifs: Implications for vaccination and antibody therapeutics. Glycobiology.

[23] Wu ZL, Whitaker M, Person AD, Kalabokis V (2020). Detecting substrate glycans of fucosyltransferases with fluorophore-conjugated fucose and methods for glycan electrophoresis. Glycobiology.

[24] Jackson P (1990). The use of polyacrylamide-gel electrophoresis for the high-resolution separation of reducing saccharides labelled with the fluorophore 8-aminonaphthalene-1,3,6-trisulphonic acid. Detection of picomolar quantities by an imaging system based on a cooled charge-coupled device. Biochem J.

[25] Savicheva EA (2021). Fluorescence assisted capillary electrophoresis of glycans enabled by the negatively charged auxochromes in 1-aminopyrenes. Angew. Chem. Int. Ed. Engl..

[26] Wu ZL (2019). Direct fluorescent glycan labeling with recombinant sialyltransferases. Glycobiology.

[27] Weinstein J, Lee EU, McEntee K, Lai PH, Paulson JC (1987). Primary structure of beta-galactoside alpha 2,6-sialyltransferase. Conversion of membrane-bound enzyme to soluble forms by cleavage of the NH2-terminal signal anchor. J. Biol. Chem..

[28] Qi F (2020). ST3GAL3, ST3GAL4, and ST3GAL6 differ in their regulation of biological functions via the specificities for the alpha2,3-sialylation of target proteins. FASEB J..

[29] Okajima T (1999). Molecular cloning of a novel alpha2,3-sialyltransferase (ST3Gal VI) that sialylates type II lactosamine structures on glycoproteins and glycolipids. J. Biol. Chem..

[30] Shi X, Jarvis DL (2007). Protein N-glycosylation in the baculovirus-insect cell system. Curr. Drug Targets.

[31] Antonopoulos A (2020). Site-specific characterisation of SARS-CoV-2 spike glycoprotein receptor binding domain. Glycobiology.

[32] Goda HM (2008). Molecular cloning, expression, and characterization of a novel endo-alpha-N-acetylgalactosaminidase from *Enterococcus faecalis*. Biochem. Biophys. Res. Commun..

[33] Yeh JC, Ong E, Fukuda M (1999). Molecular cloning and expression of a novel beta-1, 6-N-acetylglucosaminyltransferase that forms core 2, core 4, and I branches. J. Biol. Chem..

[34] Amado M, Almeida R, Schwientek T, Clausen H (1999). Identification and characterization of large galactosyltransferase gene families: Galactosyltransferases for all functions. Biochim. Biophys. Acta.

[35] O’Shea M, Samuel M, Konik C, Morell M (1998). Fluorophore-assisted carbohydrate electrophoresis (FACE) of oligosaccharides: Efficiency of labelling and high-resolution separation. Carbohyd. Res..

[36] Pardi N, Hogan MJ, Porter FW, Weissman D (2018). mRNA vaccines—a new era in vaccinology. Nat. Rev. Drug Discov..

